# Automatic Manipulation of Magnetically Actuated Helical Microswimmers in Static Environments

**DOI:** 10.3390/mi9100524

**Published:** 2018-10-16

**Authors:** Jia Liu, Tiantian Xu, Chenyang Huang, Xinyu Wu

**Affiliations:** 1 Guangdong Provincial Key Laboratory of Robotics and Intelligent System, Shenzhen Institutes of Advanced Technology, Chinese Academy of Sciences, Shenzhen 518055, China; jia.liu1@siat.ac.cn (J.L.); cy.huang@siat.ac.cn (C.H.); xy.wu@siat.ac.cn (X.W.); 2 CAS Key Laboratory of Human-Machine Intelligence-Synergy Systems, Shenzhen Institutes of Advanced Technology, Shenzhen 518055, China; 3 University of Chinese Academy of Sciences, Beijing 100049, China; 4 Shenzhen Key Laboratory of Minimally Invasive Surgical Robotics and System, Shenzhen Institutes of Advanced Technology, Chinese Academy of Sciences, Shenzhen 518055, China; 5 Department of Mechanical and Automation Engineering, The Chinese University of Hong Kong, Hong Kong, China

**Keywords:** helical microswimmer, magnetic actuation, path planning, path following

## Abstract

Electromagnetically actuated microswimmers have been widely used in various biomedical applications due to their minor invasive traits and their easy access to confined environments. In order to guide the microswimmers autonomously towards a target, an obstacle-free path must be computed using path planning algorithms, meanwhile a motion controller must be formulated. However, automatic manipulations of magnetically actuated microswimmers are underdeveloped and still are challenging topics. In this paper, we develop an automatic manipulation system for magnetically actuated helical microswimmers in static environments, which mainly consists of a mapper, a path planner, and a motion controller. First, the mapper processes the captured image by morphological transformations and then labels the free space and the obstacle space. Second, the path planner explores the obstacle-free space to find a feasible path from the start to the goal by a global planning algorithm. Last, the motion controller guides the helical microswimmers along the desired path by a closed-loop algorithm. Experiments are conducted to verify the effectiveness of the proposed automatic manipulation. Furthermore, our proposed approach presents the first step towards applications of microswimmers for targeted medical treatments, such as micromanipulation, targeted therapy, and targeted drug delivery.

## 1. Introduction

Untethered microswimmers can be potentially used in multiple biomedical applications such as targeted drug delivery [1,2], biosensing [3], micromanipulation [4,5,6] and assembling tasks [7,8]. Such promising applications urge researches to develop automatic manipulation for magnetically actuated microswimmers. For in vivo biomedical applications, microswimmers need to find an obstacle-free path and make themselves along the planned path, to accomplish the tasks of targeted therapy and targeted drug delivery. For manual operation, this would allow for considerable time and skilled operators. The manual control method is an open-loop teleoperation without any feedback. Related studies have been reported in the literature for different operation spaces, such as operation in a microfluidic chip [9,10], in 3D space [11], and in vivo operation [12]; and for different driving methods, such as bacteria-driven operation [13] and chemically driven operation [14]. The manual control method is not appropriate for tasks that require high repetition with high precision [15].

However, developing algorithms for microswimmer automation in general is still an open and challenging topic of research. Belharet et al. [16] employed the gradient of the fast marching method (FMM) to extract the minimal paths in 2D scenarios using magnetic resonance imaging (MRI)-based predictive control. The main drawback of MRI-based navigation is the strong limitation on the magnetic gradient amplitude of MRI devices. Charreyron et al. [17] used the anytime dynamic variation of the A* algorithm to find an obstacle-free path in environments containing crystals. Jing et al. [18] implemented an A* algorithm with a suitable cost function to ensure that the microrobot can avoid obstacles with minimum turns in the path. Li et al. [19] described a smart microvehicle for precise autonomous navigation in complicated environments and traffic scenarios using D* lite and a fuzzy logic controller. But the discrete-based algorithms, A* and D* lite, need a grid map to label the obstacle space and obstacle-free space. This is a memory-consuming task. Ju et al. [20] developed a static path planner based on the rapidly-exploring random tree (RRT) algorithm to generate collision-free paths for cell transportation. Kim et al. [21] employed the feasible path planner called RRT and a feedback control scheme to guide the cell to a desired position and orientation. Kim et al. [22] used gradient path planning to make the microrobot choose the minimal cost path between the two diverged ways. However, cluttered environments are often not taken into consideration. Temel et al. [23] controlled a mini swimmer inside fluid filled Y- and T-shaped channels by changing magnetic rotation rates. Barbot et al. [24] manipulated a helical microrobot in a microfluidic chip and showed its three different motions. Liu et al. [25] proposed an electromagnetic manipulation system for automatically controlling the locomotion of microrobots. However, these are absent from efficient path planning algorithms.

Various control schemes for magnetically actuated microswimmers have been reported in the past few years. Xu et al. [26] identified the helical propulsion dynamic model by experiments. Mahoney et al. [27] developed an open-loop control scheme for magnetically actuated helical microswimmers and showed its U-turn ability. Fu et al. [28] optimized helical geometries and set up a linear response between the velocity and frequency. Tottori et al. [29] conducted experiments about the carrying of spherical objects in 3D space. However, these sorts of studies employed open-loop control schemes, which are subject to external disturbances. The literature on closed-loop control for microswimmers has also been presented. Xu et al. [30] implemented planar path following based on 3D steering scaled-up helical microswimmers. Guan et al. [31] employed closed-loop control to complete arbitrary planar path following. They employed the mode-free method to design the control law, which gains satisfactory control results.

In this work, we present an automatic manipulation system, including self-positioning, path planning and following control of a helical microswimmer using a Helmholtz coil system in cluttered environments. The automatic manipulation system developed in this paper employs an efficient planning algorithm called optimal rapidly-exploring random tree (RRT*), takes cluttered environments into consideration, and uses a closed-loop control algorithm to guide the microswimmer from the star to the goal. Related works used the discrete-based algorithms, such as A* and D*, or used the basic sampling-based algorithm RRT. For A* and D*, their shortcomings are consuming more memory to store the map information, as well as not finding a path through narrow passages. For RRT, it is able to find a suboptimal solution when its searching time is infinite. To improve its search efficiency, RRT* is implemented for magnetically actuated microswimmers. The experimental results show the effectiveness of the proposed automatic manipulation system.

The remainder of this paper is organized as follows. Section 2 gives a brief introduction on the automatic manipulation system. Section 3 presents the fundamental theory of global path planners. Section 4 develops a motion controller for path following tasks of magnetically actuated helical microswimmers. Section 5 introduces the experiment system and Section 6 shows the results and analysis. Finally, Section 7 concludes the paper, and presents the perspectives of future work.

## 2. Automatic Manipulation System Architecture

**Figure 1:**
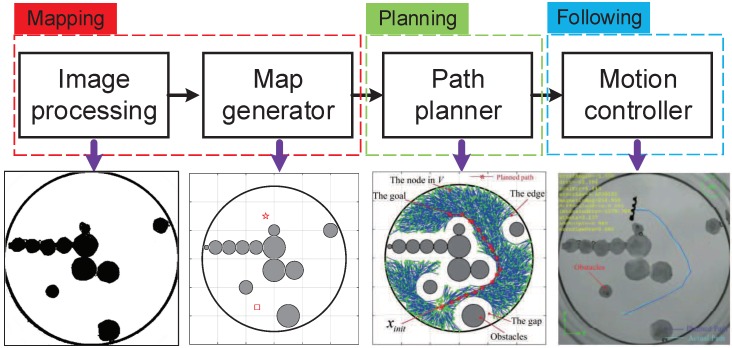
Schematic of automatic manipulation system for helical microswimmers: mapping, planning, and following. First, the map is determined via image morphological transformations. After the free space and the obstacle space are labeled, a global path in the obstacle-free space is generated by a path planner. Last, the path following controller makes the barycenter of helical microswimmers converge on the planned path.

The automatic manipulation system architecture, as shown in Figure 1, contains three subsystems: the first part is mapping; the second part is path planning in cluttered environments; the third part is path following.

The mapper includes two modules: (a) a vision capturing module to measure the environment, and (b) an image processing module to detect the obstacle-free space via image morphological transformations. The free space and the obstacle space are labeled on the map. The path planner includes one functional module, a path planning module for generating a global path in the obstacle-free space. The motion controller includes one functional module, a path following controller pilots the helical microswimmer and makes sure its barycenter converges on the planned path by the path planner.

The following section will discuss how to plan the path for magnetically actuated helical microswimmers in detail.

## 3. Static Path Planning

Current path planning algorithms have demonstrated their effectiveness in generating trajectories automatically while avoiding obstacles. While these approaches have mainly been used for macroswimmers, not too many of them have been effectively applied to magnetically actuated macroswimmers. There are two common planning methods: discrete-based algorithms and sampling-based algorithms. Discrete-based algorithms, for example the A* and D* algorithms, require a set grid resolution in order to find a least-cost path. Li et al. [19] had proved that an autonomous navigation system could guide the vehicle movement in complex environments using discrete-based algorithms. The discretization of the A* and D* algorithms reduce the likelihood of finding a truly optimal solution and are not able to find paths through narrow passages. Whereas, the sampling-based algorithms, such as the rapidly-exploring random tree (RRT) and optimal rapidly-exploring random tree (RRT*), can overcome this shortcoming.

**Figure 2:**
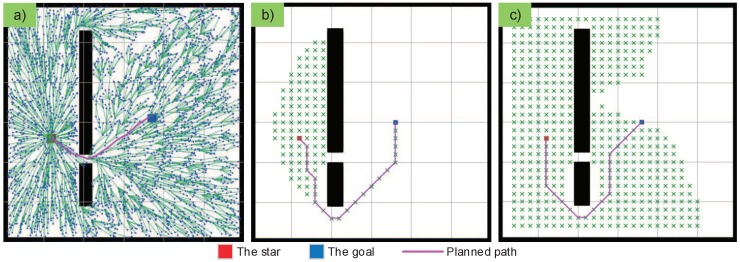
A simulation comparison of the (**a**) optimal rapidly-exploring random tree (RRT*), (**b**) A*, and (**c**) D* planners in the same scenario. A* and D* fail to find the shortest path because of the optimal path within a narrow portion, however the RRT* planner successfully finds the suboptimal path through the narrow portion.

The RRT planner is ideally suited for solving the single query problem. In our scenario, the obstacles always keep their position. The sampling-based algorithm is therefore more suitable for planning the paths of magnetically actuated macroswimmers in our experiments. Given enough samples and time, an RRT path planner is guaranteed to find a suboptimal solution whenever it exists. To improve its search efficiency, Karaman and Frazzoli [32] proposed an optimal method, RRT*, which can arrive at asymptotic optimality as more points are sampled, by rewiring the existing tree. Although the RRT* planner may not be perfect in the light of the computational cost, it shows a targeted capacity to rapidly exploring the feasible paths for magnetically actuated macroswimmers. As shown in Figure 2, A* and D* fail to find the shortest path because of the optimal path within a narrow portion, but the RRT* planner successfully finds the suboptimal path through the narrow portion.

Therefore, RRT* will be implemented for magnetically actuated microswimmers in complex environments in order to find the suboptimal path for micromanipulation, targeted therapy, and targeted drug delivery in this paper.

**Figure 3:**
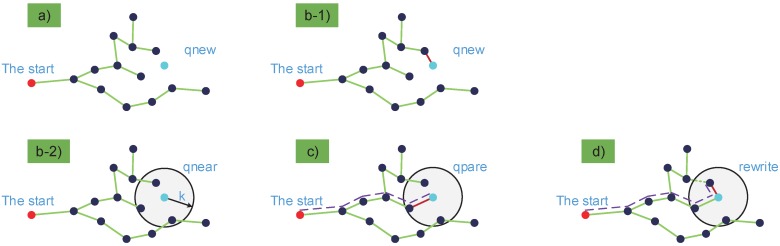
Illustration of the rapidly-exploring random tree (RRT) and RRT* algorithms. RRT consists of operations (**a**,**b-1**). RRT* consists of operations (**a**,**b-2**,**c**,**d**). (**a**) qnew denotes a new selected random node, the blue node. (**b-1**) Node qnew is connect to its nearest node in the rapidly-exploring random tree. (**b-2**) qnear denotes the near node set, as shown by the grey circular area. (**c**) qnew is connected to its qpare node which has the shortest route to the start (shown as the dotted path). (**d**) The remaining nodes in the grey circular area are rewired through qnew, if it provides the shortest path towards the start.

Figure 3 shows the algorithm principle of RRT and RRT*. The RRT planner returns a new node, qnew, and then add qnew into its exploiting tree as shown in Figure 3(b-1). It never checks if the new node has the shortest route to the start. However, the RRT* planner returns the nearest vertices within a circle of radius *k*, denoted by qnear, as shown in Figure 3(b-2). The selected node qnew is connected to a qpare node which has the shortest route to the start, as shown in Figure 3c. The remaining nodes in the grey circular area are rewired through qnew, if it provides the shortest path to the start, as shown in Figure 3d. Hence every new node in RRT* will endeavor to improve all local connections within a predefined radius.

**Figure 4:**
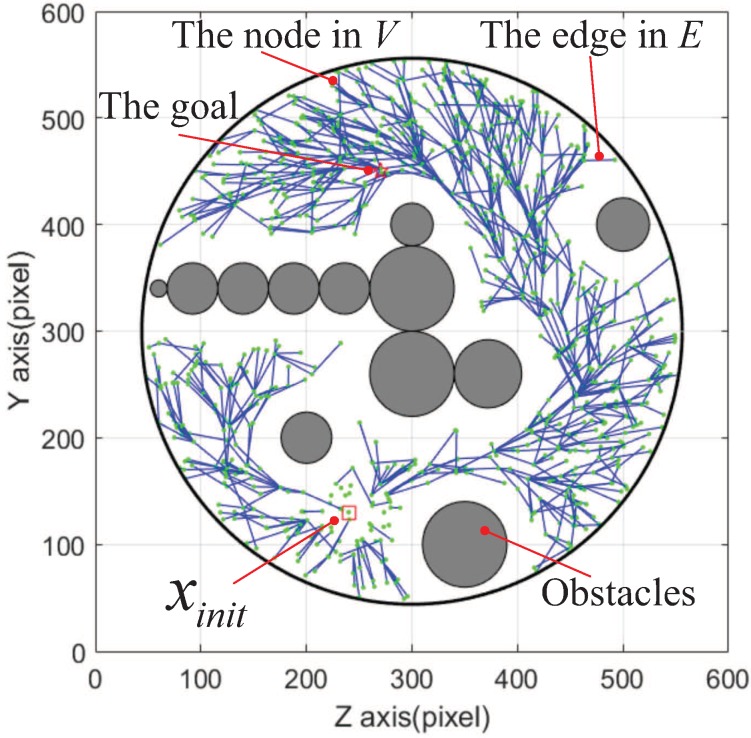
An illustration of the RRT* planner applied to our experiment. The start is denoted by a red square, the goal is denoted by a red star. The blue segments are recorded in the edge set *E*, and the green points are recorded in the state node set *V*.

For our experimental scenario, Figure 4 show the searching procedure of RRT* in the obstacle-free space. The green nodes are the random sampling points in searching procedure. The blue lines are the feasible paths for a magnetically actuated microswimmer. The path tree consists of the green nodes and blue lines. Given a tree T=(V,E), let the start node by $x_{init}$, which is denoted by a red square in Figure 4. Additionally, the goal is denoted by a red star. A brief description of RRT* can be found in Reference [32].

## 4. Motion Controller Design

**Figure 5:**
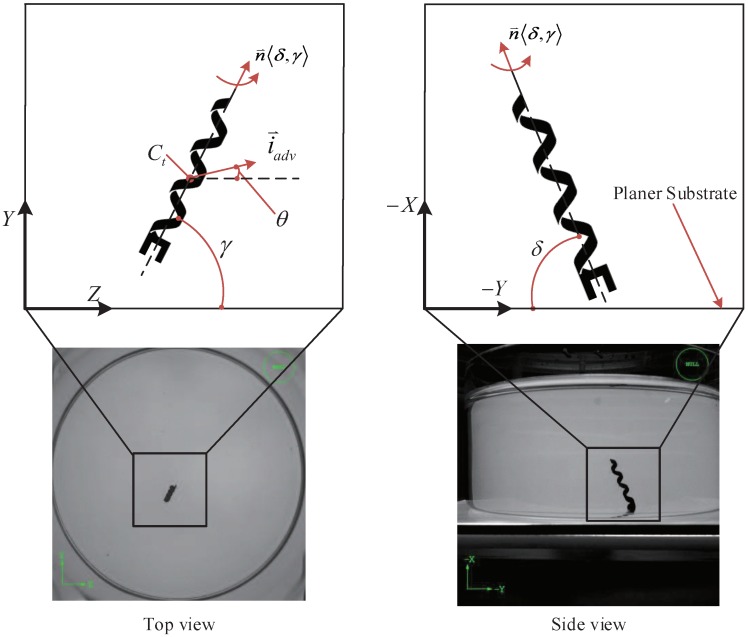
Illustrating the locomotion of helical microswimmers on a planar substrate in top-view and side-view.

Purcell [33,34] presented two efficient swimming methods: a corkscrew type rotation propulsion and an oscillating-oar-type motion. Helical microswimmers mimic the corkscrew type mode of *E. coli*, and gain propulsion by rotating their helical bodies, and steer by controlling the magnetic direction. A 3D Helmholtz magnetic coil system is used to wirelessly drive the magnetic microswimmer. A magnetic torque can be applied to the helical microswimmer to steer them along any direction in the coil workspace. In this paper, as shown in Figure 5, the head of the helical microswimmer will touch the substrate during its locomotion, because the propulsion force of microswimmer is less than the gravitational force. The coordination system is illustrated in Section 5.

In our notation, $\vec{n}\langle \delta ,\gamma \rangle$, is the rotation axis of the helical microswimmer, which consists of two key parameters δ and γ. γ is the angle between $\vec{n}$ and the *Z*-axis, and δ is the inclination angle between $\vec{n}$ and the *Y*-axis. θ is the swimming direction of the helical microswimmer, which is the angle between ${\vec{i}}_{adv}$ and the *Z*-axis. ${\vec{i}}_{adv}$ is its locomotion vector.

### 4.1. Path Following Problem

**Figure 6:**
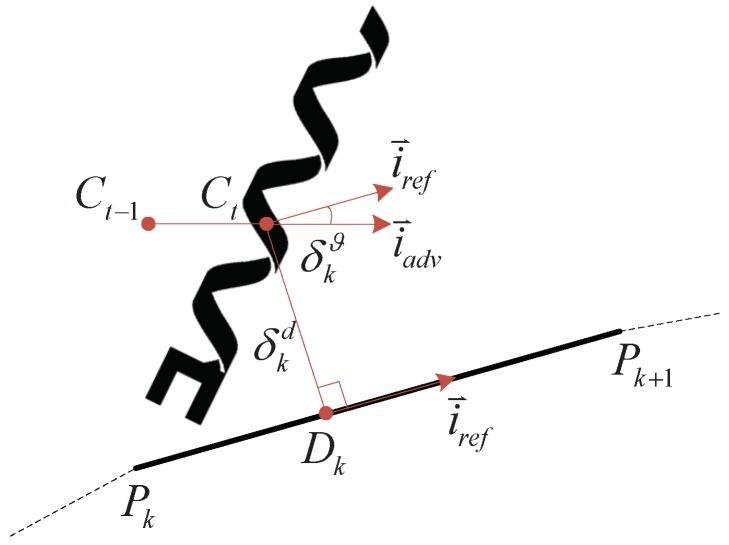
A schematic of linear segment path following.

2D planar path following can be decomposed into planar linear segment following. The short curve on a desired path can be approximated by a straight line. Thus the desired path can be described by key points belonging to approximated linear segments. So the path following problem can be changed to a linear segment following problem, as shown in Figure 6.

In Figure 6 we employ the following symbols:

$P_{k}$ and $P_{k+1}$ are the start and end points on one linear segment.
$C_{t-1}$ and $C_{t}$ are the previous and current barycenter positions of the helical microswimmer.
$D_{k}$ is the projection of $C_{t}$ onto the linear segment $P_{k}P_{k+1}$.
$\delta_{k}^{d}$ is the distance error between barycenter $C_{t}$ and projection point $D_{k}$ on the path.
${\vec{i}}_{adv}$ is the unit vector along $C_{t-1}C_{t}$
${\vec{i}}_{ref}$ is the unit vector along $P_{k}P_{k+1}$.
$\delta_{k}^{\vartheta }$ is the angle error between ${\vec{i}}_{ref}$ and ${\vec{i}}_{adv}$

$\delta_{k}^{d}$ and $\delta_{k}^{\vartheta }$ can be calculated by simple math operations in the image processing. The path following controller is designed to drive the distance error $\delta_{k}^{d}$ and angle error $\delta_{k}^{\vartheta }$ to a threshold $\varepsilon =[\varepsilon_{d},\varepsilon_{\vartheta }]$. When both the distance error $\delta_{k}^{d}$ and angle error $\delta_{k}^{\vartheta }$ are less than the threshold ε, the path following controller stops updating the control law on the current segment.

### 4.2. Motion Controller Design

In the control scheme, the dynamic model of the helical microswimmer and external disturbances are lumped together and treated as a generalized disturbance. This can eliminate the complex dynamics modeling of the helical microswimmer due to hydrodynamics and un-modeling force between the head and the substrate. Thus, a model-free control scheme is employed in this paper, such as proportional–integral–derivative (PID) controller. It is a closed-loop mechanism, which has been widely used in various robot systems and other industrial applications.

To formulate the error, a scaled error E(t) is present to indicate the error component:

(1) $$E\left(t\right)=\frac{\delta_{k}^{d}}{\delta_{k}^{d}+\delta_{k}^{\vartheta }}\delta_{k}^{d}+\frac{\delta_{k}^{\vartheta }}{\delta_{k}^{d}+\delta_{k}^{\vartheta }}\delta_{k}^{\vartheta }.$$

**Figure 7:**
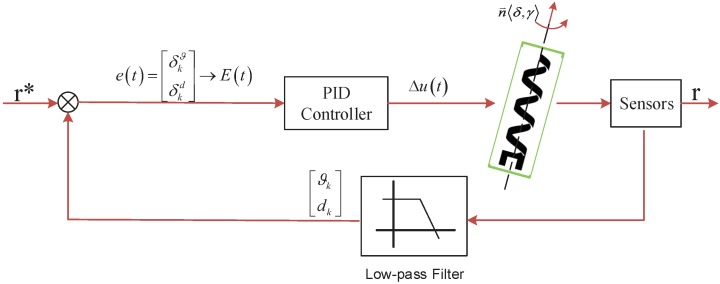
Block diagrams of the 2D path following control.

A larger $\delta_{k}^{d}$ tends to steer the microswimmer to move towards the path preferentially based on $\delta_{k}^{d}$. On the contrary, a larger $\delta_{k}^{\vartheta }$ tends to steer the microswimmer to converge to the path preferentially based on $\delta_{k}^{\vartheta }$. The scaled error E(t) can be measured in the image frame. It only needs to make E(t) to converge the error interval and the microswimmer can follow the desired path. The block diagram of the path following control is shown in Figure 7.

(2) $$\Delta u\left(t\right)=K_{p}E\left(t\right)+K_{I}\int E\left(t\right)dt$$

where $K_{p}$, $K_{I}$, all non-negative, denote the coefficients for the proportional and integral terms respectively. The target direction angle $\gamma^{*}$ can be calculated by the real-time direction angle γ and the output of the PID controller $\Delta u\left(t\right)$ via:

(3) $$\gamma^{*}=\gamma +\Delta u\left(t\right)$$

The target rotation direction of the helical microswimmer ${\overset{⇀}{\;n}}^{*}$ can be calculated with the target direction angle $\gamma^{*}$ and target inclination angle $\delta^{*}$. The target $\delta^{*}$ is fixed for many tasks. Equation (3) shows how to use the output Δu(t) to change the locomotion direction of the helical microswimmer. The following section will apply the direction angle γ to the magnetic field *B*.

## 5. Manipulation System

### 5.1. Helical Microswimmers

**Figure 8:**
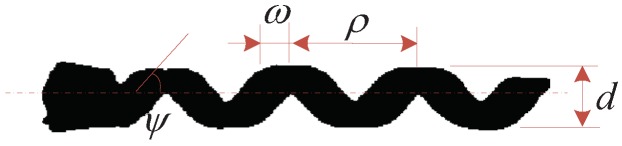
Microswimmer geometry: pitch length ρ=4 mm, diameter d=1.5 mm, ω=1.2 mm, ψ = 36.87°.

The helical microswimmer used in this research is a rigid-body with an Nd-Fe-B permanent magnet planted in its head, whose magnetization direction is perpendicular to the swimmer’s body axis. The helical swimmer was made of resin by 3d prod (http://www.3dprod.com/) [35]. The diameter *d*, pitch length ρ, and number of turns *n* of the microswimmer are 1.5 mm, 4 mm, and 3.5 turns, respectively. The helical body width *w* is 1.2 mm, as shown in Figure 8.

The helical microswimmers swim in a glycerol solution. The density and viscosity at 20°C are 1.238 g/cm^3^ and 219mPa·s [36], respectively. The locomotion velocity of the helical swimmer is 2–5 mm/s. Therefore, the calculated Reynolds number is approximately 0.011–0.028.

### 5.2. Magnetic Actuated System

**Figure 9:**
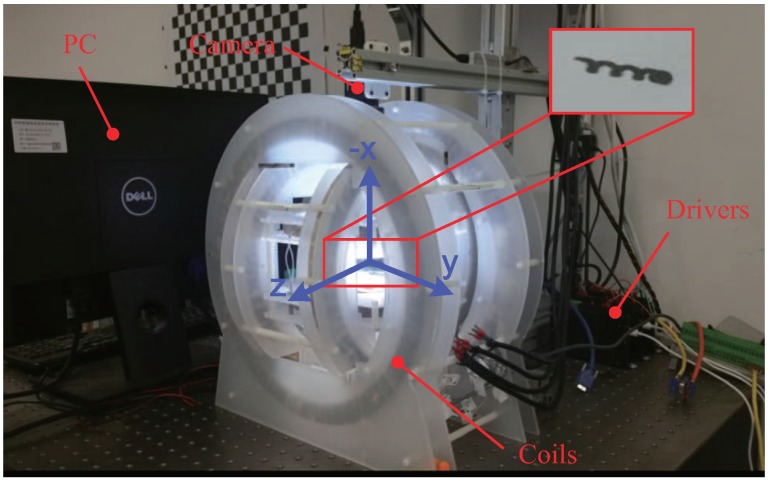
The 3D Helmholtz coil system setup.

The helical microswimmers in this paper are actuated by a 3D Helmholtz coil system, as shown in Figure 9. 3D Helmholtz coil systems have actuated different microswimmers such as rigid helical microswimmers [31] and soft helical microswimmers [37]. It can generate a uniform magnetic field in a working space with a size of approximately 80 mm × 50 mm × 40 mm, and is driven by 3 Maxon ESCON 70/10 motor drivers (Maxon Motor, Sachseln, Switzerland). A PC sends out control signals through a Sensoray S826 PCIe A/D IO card to the motor drivers (Sensoray Co., Inc. 7313 SW Tech Center Dr. Tigard, OR 97223, USA). This system also comes with a single camera (PointGreyGS3-U3-41C6M, FLIR Integrated Imaging Solutions, Inc., Richmond, BC, Canada) mounted on the top of the 3D Helmholtz coils, providing overviews for monitoring.

The magnetization of the helical swimmer is noted as M, which is perpendicular to the rotation axis $\vec{n}\langle \delta ,\gamma \rangle$. The magnetic torque *T* exerted on the helical swimmer can be expressed as:

(4) T=M×B.

The magnetic field *B* can be decomposed in the directions perpendicular to its rotation axis $B_{∥}$, and colinear to its rotation axis $B_{⊥}$ As the magnetization of the helical swimmer is always perpendicular to its own axis $\vec{n}\langle \delta ,\gamma \rangle$, the magnetic torque can also be decomposed in these two directions:

(5) $$T=M\times B_{∥}+M\times B_{⊥}.$$

The magnetic field $B_{⊥}$ yields self-rotation of the helical microswimmer and is expressed by:

(6) $$B_{⊥}=B\cos\left(2\pi ft\right)\tilde{u}+B\cos\left(2\pi ft\right)\tilde{v},$$

where $\tilde{u}$ and $\tilde{v}$ denote the unit vectors of the plane perpendicular to the self-rotation axis of the helical swimming robot [35]. The magnetic field $B_{⊥}$, which contributes to the steering, depends on the orientation error of the helical microswimmer. The orientation error can be defined by the sine value of the real-time rotation axis and the target rotation axis: $\sin\left({\overset{⇀}{\;n}}^{*}\langle \delta ,\gamma^{*}\rangle ,\overset{⇀}{\;n}\right.\langle \delta ,\gamma \rangle$).

The magnetic field $B_{∥}$ can be expressed as:

(7) $$B_{∥}=-\mathrm{sign}\left(B_{⊥}\cdot {\overset{⇀}{\;n}}^{*}\langle \delta ,\gamma^{*}\rangle \right.\cdot \lambda ∥\overset{⇀}{\;n}\langle \delta ,\gamma \rangle \times {\overset{⇀}{\;n}}^{*}\langle \delta ,\gamma^{*}\rangle ∥\overset{⇀}{\;n}\langle \delta ,\gamma \rangle ,$$

where λ is the control parameter.

The self-rotation direction of the helical microswimmer $\overset{⇀}{\;n}\langle \delta ,\gamma \rangle$ can be expressed by:

(8) $$\overset{⇀}{\;n}\langle \delta ,\gamma \rangle =\left[\sin\left(\delta \right),\cos\left(\delta \right)\sin\left(\gamma \right),\cos\left(\delta \right)\cos\left(\gamma \right)\right].$$

$B_{∥}$ can be adjusted by δ and γ. Figure 8 shows the adjustment procedure of the self-rotation direction $\vec{n}\langle \delta ,\gamma \rangle$. The desired direction angle $\gamma^{*}$ can be calculated by the output of Equation (3). In the following, the adjustment of the locomotion direction for magnetically actuated helical microswimmers is shown in detail.

The automatic manipulation of the magnetically actuated helical microswimmer is shown as follows. First the navigation for the magnetically actuated helical microswimmers proceeds via:

Step 1: Get the map via image morphological transformations.

Step 2: Generate the planned trajectory of the helical microswimmer via informed RRT* on the map.

Step 3: Run the closed-loop control algorithm.

The closed-loop control algorithm in this paper is described as follows:

Step 1: Generate the desired trajectory of the microswimmer through the control software or the planned path by RRT*. The planned path consists of linear segments.

Step 2: Select the region of interest (ROI) and locate the center of the microswimmer.

Step 3: According to position feedback, calculate the desired coil current based on the established control algorithm. The swimming direction of the microswimmer is given by the direction angle γ. The inclination angle δ is fixed for many tasks. As shown in Figure 7, the output $\Delta u\left(t\right)$ of the controller is calculated based on the feedback error and the microswimmer converges on the reference path using Equation (3).

Step 4: Set the calculated coil current to the Maxon motor drivers by the PC, then generate the desired magnetic rotation direction in the workspace, so that the microswimmer can be controlled.

## 6. Experiments

The feasibility of the automatic manipulation system developed in this paper will be verified. The helical microswimmer used in the experiments is shown in Figure 8 and experiments were performed by the setup shown in Figure 9. First of all, the parameters of the motion controller should be determined. Compensating for the trade-off between the accuracy of closed-loop control and the response speed of the microswimmer, the sampling period of the controller was chosen as 0.06 s (the control frequency is about 16 Hz). The helical microswimmer has an inclination angle of 45° and touches the substrate, which is within the working space of the 3D Helmholtz coils. The control parameters were chosen as $K_{p}=0.8$, $K_{I}=1.2$. Under the step-out frequency of the helical microswimmers, it could rotate synchronously with the actuation field. So the rotation frequency of the actuation field is chosen as 5 Hz in the experiments. All parameters are listed in Table 1.

**Figure 10:**
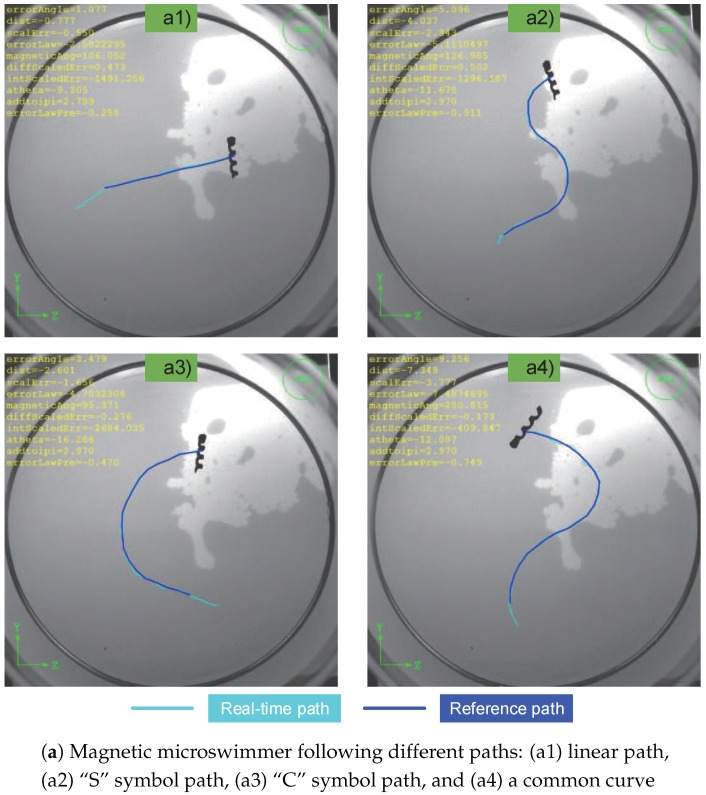

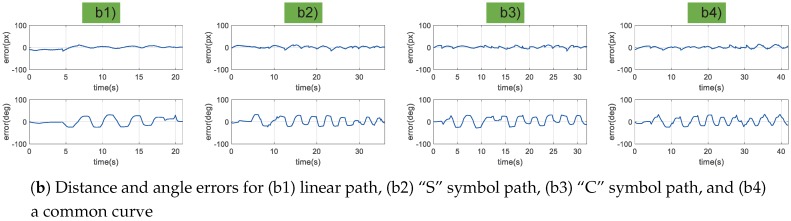
Results of 2D path following are shown in figure (**a**). The respective distance and steering angle errors are shown in figure (**b**). The reference path is denoted by a navy blue line and the real-time path is denoted by light blue.

In order to evaluate the motion controller, various paths were tested and their distance error and steering angle error are shown in Figure 10. The target is to steer the barycenter of the helical microswimmer along desired path. Figure 10(a1) shows the trajectory of the helical mircroswimmer following a straight line where the reference path is denoted by a navy blue line and the real-time path is denoted by light blue. Using the designed controller, the microswimmer first moves to the path start and then moves along the path. Figure 10(a2) and Figure 10(a3) show that the microswimmer can follow an “S” symbol path and a “C” symbol path, respectively. In addition, a common curve is tested in Figure 10(a4).

In Figure 10b, the root mean square (RMS) errors of the distance are 9.4, 8.0, 8.2, and 8.1 pixels, respectively, and the ratio κ between the distance errors of RMS and the characteristic length (350 pixels in capture image (2048 × 2048)) are 2.69%, 2.29%, 2.31%, and 2.23%, as listed in Table 2 (In this paper, 1 pixel = 0.042 mm). Although the errors tend to increase when the path turns suddenly, the proposed control scheme can steer the microswimmer to follow the desired path within a tolerant error interval. Due to the time-independence path following, the information about velocity can be overlooked.

In experiments, large path following errors will happen, as shown in Figure 10(a4). The reason is that the un-modeling force between the head and the substrate makes the helical microswimmers move off the reference path. Whereas the formulated control law can steer the microswimmer to approach to the desired path step by step.

**Figure 11:**
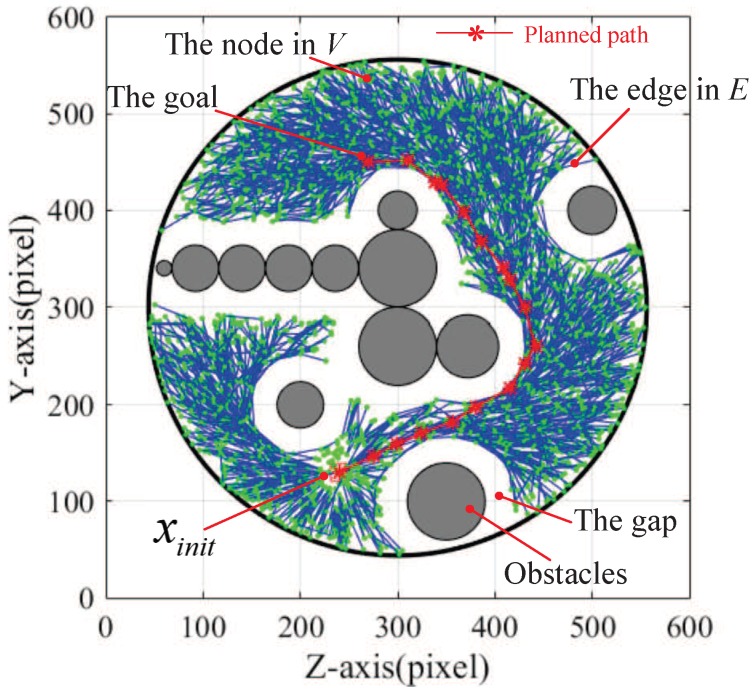
The planned path by RRT*. The red line denotes the planned sub-optimal path.

Based on the motion controller, a navigation experiment was also conducted. Figure 11 shows the rapidly Exploring random tree, T, after 5000 iterations. The red line denotes the planned sub-optimal path. The blank gaps between the edge set and the obstacles are artificially set to account for the volume of the helical microswimmer.

**Figure 12:**
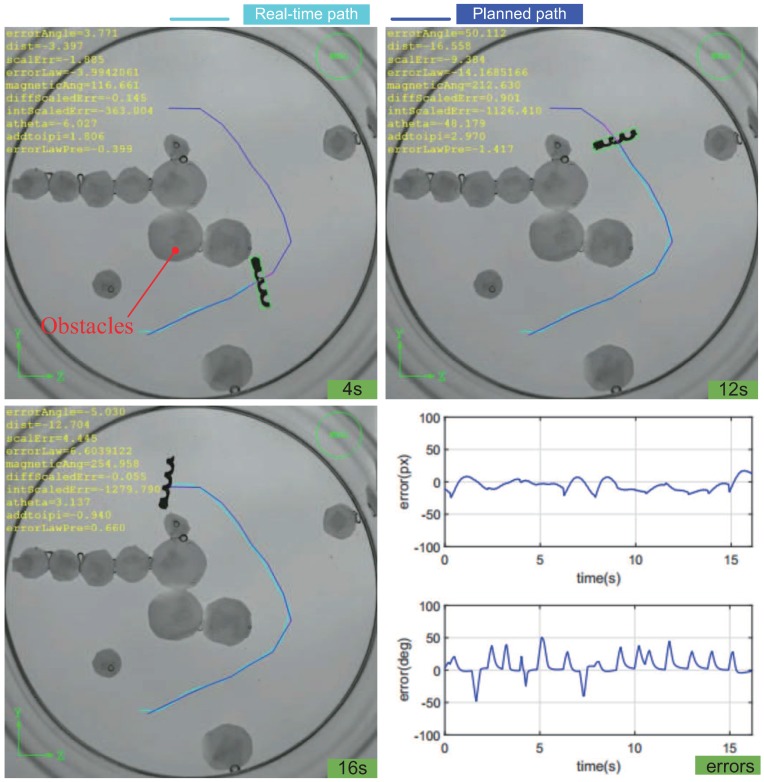
Control of the helical microswimmer on the planned path at different times.

The path planning algorithm is implemented via MATLAB off-line (2016b, MathWorks, Inc., Natick, MA, USA). For experiments, Figure 12 shows the path following procedure at different times, the following distance, and steering angle errors. The RMS of the distance error is listed in Table 1.

A detailed recording of the tasks mentioned above can be found in the Video S1: Automatic Manipulation.

## 7. Conclusions

This paper presents an automatic manipulation system, consisting of a mapper, a planner and a motion controller, for magnetically actuated helical microswimmers. First, the mapper processes the captured image by morphological transformations and then labels the free space and the obstacle space. Second, a simple model-free control scheme is developed for helical microswimmers moving on a substrate. Supplementary experiments show that the helical microswimmer successfully moves along different forms of paths. Last, a global path planner called RRT* is implemented to guide helical microswimmers to the desired goal.

In all, the automatic manipulation system developed in this paper employs an efficient planning algorithm (RRT*), takes cluttered environments into consideration, and uses a closed-loop control algorithm to guide the microswimmer from the star to the goal. Such an automatic manipulation system is the first step to autonomous manipulation in complex settings. One possible application is to carry on micromanipulation and assembling tasks. In addition, the control of the helical microswimmer presented here is dedicated to conveying a micro-device in the human body such as vessels and eyeballs. Another possible application is to locate lesions in stenosed blood vessels or eyeballs and treat them either chemically or pharmacologically by targeted drug delivery.

However, the automatic manipulation system also has such limitations as it only takes a static environment into consideration and off-line path planning. In future work, we will (1) employ learning control methods to set up a kinetic model as well as extend to the 3D path planning and path following problem, and (2) develop an online path planner and real-time path planner in dynamic environments.

## Figures and Tables

**Table 1:** Parameters in the experiments.

Parameters	Control Frequency (Hz)	Inclination Angle (°)	$K_{p}$	$K_{I}$	Rotation Frequency (Hz)
**Values**	16	45	0.8	1.2	5

**Table 2:** Root mean square (RMS) error of the distance.

Path	Linear	“S” Symbol	“C” Symbol	Curve	Planned
**RMS/pixel**	9.4	8.0	8.2	8.1	7.8
**RMS/mm**	0.395	0.336	0.344	0.340	0.328
**κ**	2.69%	2.29%	2.34%	2.31%	2.23%

## Supplementary Materials

The following are available online at http://www.mdpi.com/2072-666X/9/10/524/s1. Video S1: Automatic Manipulation.

## References

[1] Nelson B.J., Kaliakatsos I.K., Abbott J.J. (2010). Microrobots for Minimally Invasive Medicine. Annu. Rev. Biomed. Eng..

[2] Peyer K.E., Zhang L., Nelson B.J. (2013). Bio-inspired magnetic swimming microrobots for biomedical applications. Nanoscale.

[3] Han K., Shields C.W., Velev O.D. (2018). Engineering of self-propelling microbots and microdevices powered by magnetic and electric fields. Adv. Funct. Mater..

[4] Chen H., Sun D. (2012). Moving Groups of Microparticles Into Array with a Robot–Tweezers Manipulation System. IEEE Trans. Robot..

[5] Ghanbari A., Chang P.H., Nelson B.J., Choi H. (2014). Magnetic actuation of a cylindrical microrobot using time-delay-estimation closed-loop control: Modeling and experiments. Smart Mater. Struct..

[6] Martínez-Pedrero F., Tierno P. (2018). Advances in colloidal manipulation and transport via hydrodynamic interactions. J. Colloid Interface Sci..

[7] Huang T.Y., Qiu F., Tung H.W., Peyer K., Shamsudhin N., Pokki J., Zhang L., Chen X.B., Nelson B., Sakar M. (2014). Cooperative manipulation and transport of microobjects using multiple helical microcarriers. RSC Adv..

[8] Niu R., Palberg T. (2018). Seedless assembly of colloidal crystals by inverted micro-fluidic pumping. Soft Matter.

[9] Wang J., Jiao N., Tung S., Liu L. (2014). Magnetic microrobot and its application in a microfluidic system. Robot. Biomim..

[10] Katuri J., Caballero D., Voituriez R., Samitier J., Sanchez S. (2018). Directed Flow of Micromotors through Alignment Interactions with Micropatterned Ratchets. ACS Nano.

[11] Diller E., Sitti M. (2014). Three-Dimensional Programmable Assembly by Untethered Magnetic Robotic Micro-Grippers. Adv. Funct. Mater..

[12] Servant A., Qiu F., Mazza M., Kostarelos K., Nelson B.J. (2015). Controlled in vivo swimming of a swarm of bacteria-like microrobotic flagella. Adv. Mater..

[13] Park B.W., Zhuang J., Yasa O., Sitti M. (2017). Multifunctional bacteria-driven microswimmers for targeted active drug delivery. ACS Nano.

[14] Simmchen J., Katuri J., Uspal W.E., Popescu M.N., Tasinkevych M., Sánchez S. (2016). Topographical pathways guide chemical microswimmers. Nat. Commun..

[15] Xu T., Yu J., Yan X., Choi H., Zhang L. (2015). Magnetic actuation based motion control for microrobots: An overview. Micromachines.

[16] Belharet K., Folio D., Ferreira A. Endovascular navigation of a ferromagnetic microrobot using MRI-based predictive control. Proceedings of the 2010 IEEE/RSJ International Conference on Intelligent Robots and Systems (IROS).

[17] Charreyron S., Pieters R.S., Tung H.W., Gonzenbach M., Nelson B.J. Navigation of a rolling microrobot in cluttered environments for automated crystal harvesting. Proceedings of the 2015 IEEE International Conference of Intelligent Robots and Systems (IROS).

[18] Jing W., Chowdhury S., Guix M., Wang J., An Z., Johnson B.V., Cappelleri D.J. (2018). A Microforce-Sensing Mobile Microrobot for Automated Micromanipulation Tasks. IEEE Trans. Automat. Sci. Eng..

[19] Li T., Chang X., Wu Z., Li J., Shao G., Deng X., Qiu J., Guo B., Zhang G., He Q. (2017). Autonomous collision-free navigation of microvehicles in complex and dynamically changing environments. ACS Nano.

[20] Ju T., Liu S., Yang J., Sun D. (2014). Rapidly exploring random tree algorithm-based path planning for robot-aided optical manipulation of biological cells. IEEE Trans. Autom. Sci. Eng..

[21] Kim D.H., Brigandi S., Julius A.A., Kim M.J. Real-time feedback control using artificial magnetotaxis with rapidly-exploring random tree (RRT) for Tetrahymena pyriformis as a microbiorobot. Proceedings of the 2011 IEEE International Conference on Robotics and Automation (ICRA).

[22] Kim H., Cheang U.K., Rogowski L.W., Kim M.J. (2018). Motion planning of particle based microrobots for static obstacle avoidance. J. Micro-Bio Robot..

[23] Temel F.Z., Bezer A.E., Yesilyurt S. Navigation of mini swimmers in channel networks with magnetic fields. Proceedings of the 2013 IEEE International Conference on Robotics and Automation (ICRA).

[24] Barbot A., Decanini D., Hwang G. (2016). On-chip microfluidic multimodal swimmer toward 3D navigation. Sci. Rep..

[25] Wang J., Jiao N., Tung S., Liu L. (2016). Automatic path tracking and target manipulation of a magnetic microrobot. Micromachines.

[26] Xu T., Hwang G., Andreff N., Regnier S. (2014). Modeling and Swimming Property Characterizations of Scaled-Up Helical Microswimmers. IEEE-ASME Trans..

[27] Mahoney A., Sarrazin J., Bamberg E., Abbott J. (2011). Velocity Control with Gravity Compensation for Magnetic Helical Microswimmers. Adv. Robot..

[28] Fu H.C., Jabbarzadeh M., Meshkati F. (2015). Magnetization directions and geometries of helical microswimmers for linear velocity-frequency response. Phys. Rev. E.

[29] Tottori S., Zhang L., Qiu F., Krawczyk K.K., Francoobregón A., Nelson B.J. (2012). Magnetic helical micromachines: fabrication, controlled swimming, and cargo transport. Adv. Mater..

[30] Xu T., Hwang G., Andreff N., Régnier S. (2015). Planar Path Following of 3-D Steering Scaled-Up Helical Microswimmers. IEEE Trans. Robot..

[31] Guan Y., Xu T., Liu J., Xinyu W. Image-based visual servoing of helical microswimmers for arbitrary planar path following at low reynolds numbers. Proceedings of the 2017 IEEE/RSJ International Conference on Intelligent Robots and Systems (IROS).

[32] Karaman S., Frazzoli E. (2011). Sampling-based algorithms for optimal motion planning. Int. J. Robot. Res..

[33] Purcell E.M. (1977). Life at low Reynolds-number. Am. J. Phys..

[34] Purcell E.M. (1997). The Efficiency of Propulsion by a Rotating Flagellum. Proc. Natl. Acad. Sci. USA.

[35] Xu T., Hwang G., Andreff N., Regnier S. Characterization of three-dimensional steering for helical swimmers. Proceedings of the IEEE International Conference on Robotics and Automation.

[36] Hunter E.E., Brink E.W., Steager E.B., Kumar V. (2018). Toward Soft Micro Bio Robots for Cellular and Chemical Delivery. IEEE Robot. Automat. Lett..

[37] Liu J., Xu T., Guan Y., Yan X., Ye C., Wu X. (2017). Swimming Characteristics of Bioinspired Helical Microswimmers Based on Soft Lotus-Root Fibers. Micromachines.

